# Requirement of the Dynein-Adaptor Spindly for Mitotic and Post-Mitotic Functions in *Drosophila*

**DOI:** 10.3390/jdb6020009

**Published:** 2018-03-30

**Authors:** Giuliana D. Clemente, Matthew R. Hannaford, Hamze Beati, Katja Kapp, Jens Januschke, Eric R. Griffis, Hans-Arno J. Müller

**Affiliations:** 1 Division of Cell and Developmental Biology, School of Life Sciences, University of Dundee, Dundee DD1 5EH, UK; giuliana.clemente@manchester.ac.uk (G.D.C.); m.r.hannaford@dundee.ac.uk (M.R.H.); hamze.beati@uni-kassel.de (H.B.); j.januschke@dundee.ac.uk (J.J.); 2 Institut für Biologie, Fachgebiet Entwicklungsgenetik, Universität Kassel, Heinrich-Plett Str. 40, 34321 Kassel, Germany; k.kapp@uni-kassel.de; 3 Centre of Gene Regulation and Expression, School of Life Sciences, University of Dundee, Dundee DD1 5EH, UK; ergriffis@gmail.com

**Keywords:** *Drosophila*, mitosis, cell migration, mitotic spindle, Dynein

## Abstract

Spindly was originally identified as a specific regulator of Dynein activity at the kinetochore. In early prometaphase, Spindly recruits the Dynein/Dynactin complex, promoting the establishment of stable kinetochore-microtubule interactions and progression into anaphase. While details of Spindly function in mitosis have been worked out in cultured human cells and in the *C. elegans* zygote, the function of Spindly within the context of an organism has not yet been addressed. Here, we present loss- and gain-of-function studies of Spindly using transgenic RNAi in *Drosophila*. Knock-down of Spindly in the female germ line results in mitotic arrest during embryonic cleavage divisions. We investigated the requirements of Spindly protein domains for its localisation and function, and found that the carboxy-terminal region controls Spindly localisation in a cell-type specific manner. Overexpression of Spindly in the female germ line is embryonic lethal and results in altered egg morphology. To determine whether Spindly plays a role in post-mitotic cells, we altered Spindly protein levels in migrating cells and found that ovarian border cell migration is sensitive to the levels of Spindly protein. Our study uncovers novel functions of Spindly and a differential, functional requirement for its carboxy-terminal region in *Drosophila*.

## 1. Introduction

In early *Drosophila* embryogenesis, nuclei divide in a common cytoplasm without intervening cytokinesis. The nuclei divide synchronously in rapid mitotic cycles oscillating between S- and M-phases that are supported by maternal-derived gene products [1,2]. As for an archetypal cell cycle, entry into mitosis is driven by the activation of Cdk1-Cyclin-B complexes, whereas mitotic exit is accompanied by the timely inactivation of these complexes in a wave that propagates from the spindle poles towards the metaphase plate [3,4]. In most cell types, mitotic exit is under the strict control of the Spindle Assembly Checkpoint (SAC), which halts the progression into anaphase by inhibiting the activity of the E3 ubiquitin ligase APC/C until each chromosome is stably attached to the fibres of the mitotic spindle [5,6,7]. Once all of the chromosomes have achieved bi-orientation, the SAC is inactivated by the Dynein-dependent transport of key SAC components, such as Mad1 and Mad2, from the outer kinetochores [8,9,10,11,12,13]. Mitotic control and SAC regulation in *Drosophila* syncytial embryos are unique. In this system, mitosis is very robust and it does not need the SAC to ensure faithful chromosome segregation [14,15]. Indeed, *mad-2*-null embryos undergo mitosis, normally with very little rate of aneuploidy and develop into viable, fertile adults [14].

Despite the lack of the requirement of a SAC in normal *Drosophila* syncytial divisions, SAC activity is still crucial when microtubule attachment and chromosome alignment are compromised. Mutations in genes encoding for the checkpoint proteins Rough deal (Rod), ZW-10, Mps1, Bubr1 and Bub3 are lethal and accompanied by a high rate of aneuploidy [16,17,18,19,20]. These severe mitotic phenotypes were attributed not only to defects in the mitotic checkpoint, but also to impaired kinetochore activity. A good example is offered by the evolutionary conserved ROD-Zwilch-ZW10 (RZZ) complex [21]; the RZZ complex has a dual activity in controlling the “on-off” state of the mitotic checkpoint. First, it promotes the stable localisation of Mad1-Mad2 at kinetochores of unattached chromosomes [12,22]. Secondly, by recruiting Dynein to kinetochores, the RZZ complex regulates the establishment of stable end-on kinetochore-microtubule attachments, thereby contributing to the bilateral orientation of chromosomes at the spindle equator. Finally, it contributes to SAC silencing through the recruitment of Dynein upon microtubule-kinetochore attachment [23,24].

How does the RZZ complex localise Dynein at kinetochores? The RZZ complex not only directly interacts with the Dynactin complex and the Dynein intermediate chain, but it localises Dynein also by interaction with the Dynein co-factor Spindly [9,25,26,27,28,29]. *Drosophila* Spindly has a predicted rod-like structure that is organised in four coiled-coil domains in the amino-terminal (N-terminal) half and four positively-charged repeats in the carboxy-terminal (C-terminal) half of the protein [9]. Spindly localises to the outer kinetochore by directly interacting with the RZZ complex through its C-terminal domain [30,31,32,33]. Once bound to RZZ, Spindly extends its N-terminal half out from the kinetochore to bind Dynein [27,30,34]. In this respect, Spindly shares similarities to other Dynein-adaptor proteins, such as BicD-2 [35,36], which rely on their C-terminal domain to interact with cargoes (i.e., the kinetochore) while the N-terminal portion mediates interaction with the Dynein-Dynactin motor complex [9]. Thus, Spindly controls cell-cycle progression by linking the RZZ complex with the Dynein motor, thereby acting as a molecular bridge between chromosome bi-orientation and Dynein-dependent checkpoint silencing [27]. In addition to its role in SAC function, the phenotype of Spindly depleted S2 cells during interphase suggested a role for Spindly in the control of cell shape and the organisation of the cytoskeleton [9]. However, a function for Spindly in a tissue or an organismal context has thus far remained elusive.

Here, we analysed Spindly function in *Drosophila* using loss-of-function and gain-of-function experiments in a tissue-specific fashion. In neuroblasts, Green Fluorescent Protein (GFP)-tagged Spindly localises dynamically during mitosis and like the human protein, its localisation to the kinetochore depends on the C-terminal region. Conversely, deletion of the C-terminal region only mildly affects Spindly function in the early embryo, and it is still able to localise to the kinetochore in a dynamic fashion. Thus, *Drosophila* Spindly exhibits a differential requirement for its C-terminal region in different cell types. In addition to the mitotic defects due to the loss of function, overexpression of Spindly in the germ line resulted in eggs with altered shape, a mild ventralisation, and severely compromised embryogenesis. Finally, we demonstrate that normal levels of Spindly protein are required for migration of the follicular border cells in oogenesis. We conclude that, in addition to its functions in mitotic cells, *Drosophila* Spindly is important for the movement of post-mitotic cells.

## 2. Results

### 2.1. Requirements for Dynamic Subcellular Localisation of Spindly

**Figure 1:**
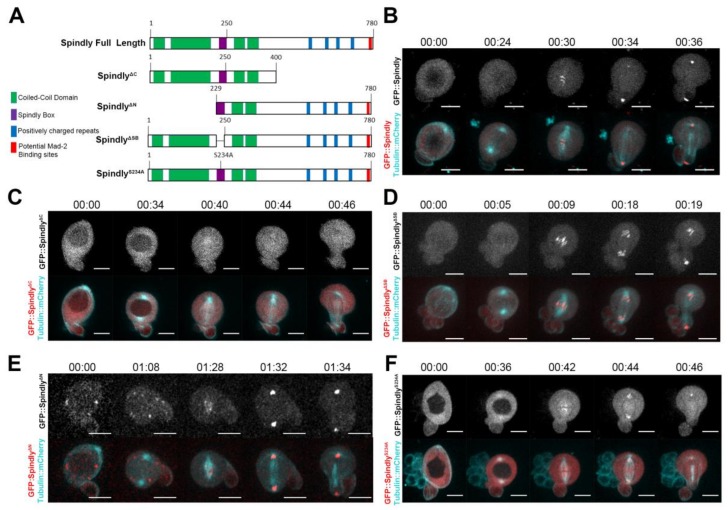
Identification of Spindly protein domain(s) required for dynamic localisation in mitosis. (**A**) Schematic drawing showing the full-length *Drosophila* Spindly protein and mutant constructs generated in this study. Spindly is a predicted coiled-coil domain containing protein (N-terminal half, in green) characterised by a positively charged C-terminal domain (in blue). The highly conserved repeat motif is depicted (Spindly box, in purple). Spindly localises to the outer kinetochore plate by interacting with the RZZ complex through its C-terminal domain [30,31,32,33]. In addition to the importance of the C-terminal half, other regions of the protein contribute to kinetochore targeting. Human Spindly requires the first coil-coiled domain, the Spindly box and a region between residues 440–564 for kinetochore binding [30]; (**B**–**F**) Representative examples of dividing neuroblasts overexpressing the indicated *UAS*>>[Green Fluorescent Protein (GFP)]::Spindly variants under the control of the *worniu*>Gal4 driver; (**B**) GFP::Spindly full length gets recruited to kinetochores at early pro-metaphase (00:26) and can be seen moving from KT to poles at anaphase (00:32). In all cases (*n* = 21) the protein localised this way; (**C**) GFP::Spindly^ΔCt^ never localised at kinetochores throughout mitosis (*n* = 43). Mutant constructs deleted of the Spindly box (GFP::Spindly^ΔSB^) (*n* = 21) (**D**); N-terminal half (*n* = 40) (**E**) or a point mutant S234A (GFP::Spindly^S234A^) (*n* = 33) (**F**) showed a localisation dynamics similar to the full-length protein. Note the speckled pattern of GFP::Spindly^ΔNt^ in interphase, which was observed in 31/40 cases. The minor differences between the distribution of the full length GFP::Spindly and the GFP::Spindly^ΔSB^ (**D**) or GFP::Spindly^S234A^ (**F**) proteins are due to small variations in the expression of the *wor*>*Gal4* driver and slight differences in the time resolution of the still images; for a full comparison, we refer to the respective movies. Time stamp: hh:min. Scale bars: 10 μm.

Spindly localises dynamically in dividing cultured cells [9,27,30]. During prophase and metaphase, Spindly associates with the kinetochore and moves poleward in early anaphase. While details of the requirement of Spindly protein domains for its localisation are available for the human homologue [30], this information is still missing for *Drosophila* Spindly. Therefore, to perform a domain-function analysis, we generated a similar range of GFP-tagged Spindly protein constructs, as studied by Barisic et al. [30] and expressed them in larval neuroblasts, using the Gal4/UAS system [37] (Figure 1A). Larval neuroblasts (NBs) are rather large cells, which can be cultured in vitro and are readily identified and imaged during mitosis at high spatial resolution [38]. For these reasons, larval NBs provide an excellent model to study the dynamic localisation of proteins during mitosis. Overexpression of GFP::Spindly in neuroblasts revealed that the protein localises to the kinetochores in a dynamic fashion during mitosis (Figuer 1B, Supplementary Materials Movie 1). A construct that lacks the C-terminal 380 amino acids did not localise to kinetochores during neuroblast division, suggesting that, like in humans and *C. elegans,* the C-terminal half is essential for normal Spindly localisation to kinetochores (Figure 1C; Supplementary Materials Movie 2). All the other constructs localised to the kinetochores during mitosis (Figure 1D–F; Supplementary Materials Movies 3–5). Another interesting observation was that in 78% of cases (*n* = 40), the protein lacking the N-terminal 228 amino acids localised to cytoplasmic punctae in interphase, which were never observed with any of the other constructs (Figure 1E; Supplementary Materials Movie 4). The speckles do not seem to be polarised in any way. With the caveat that overexpression of GFP-conjugated fusion proteins may cause the abnormal localisation of proteins in a wild type background, our results suggest that the N-terminal domain is required for the normal distribution of the protein in interphase. Furthermore, our experiments confirm that similar to human Spindly, the C-terminal domain is important for binding to the kinetochore.

### 2.2. Requirement of Spindly for Initiation of Syncytial Cleavage Division

Since Spindly knock-down by RNAi worked efficiently in *Drosophila* S2 cells [9], we selected shRNAs that were directed against *spindly* mRNA that can be expressed under temporal and spatial control by the Gal4/UAS system [26,37,39]. A maternal Gal4 driver (alpha-*tubulin67*>*Gal4*) was used to express UAS-controlled shRNA targeting *spindly* mRNA in oocytes and embryos (called *spindly*[mat67>RNAi] hereafter). The efficacy of the transgenic RNAi knockdown was assayed by immunoblotting of embryo lysates with an affinity-purified Spindly antibody [9], and a marked reduction in the levels of Spindly protein was observed at all of the temperatures tested (Supplementary Materials Figure S1).

**Figure 2:**
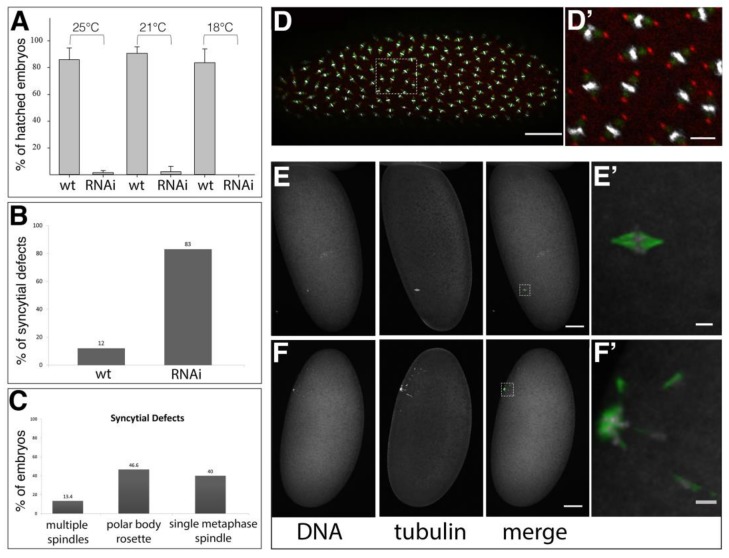
Phenotype of *mat-tub*>>*Gal4* driven Spindly knock-down. (**A**) Larval hatching rates of *spindly*[*mat67*>>RNAi] embryos at temperatures indicated (*n* = 100); for protein levels see Supplementary Materials Figure S1; (**B**) Penetrance of mitotic arrest during syncytial divisions in *spindly mat67*>>RNAi embryos (*n* = 100). (**C**) Expressivity of syncytial cleavage defects in *spindly mat67*>>RNAi embryos (*n* = 30); (**D**,**D’**) Mitotic domains in wild-type syncytial blastoderm embryo**.** Nuclei are stained with DAPI (grey), anti-beta-tubulin (green) and anti-Centrosomin (red). Scale bars represent 50 µm (**D**) and 10 µm (**D’**); (**E**,**F**) Syncytial division defects in *spindly mat67*>>RNAi embryos. The images are maximum-intensity projections of 10 μm z-stack (at spacing of 1 μm); (**E**,**E’**) Embryos often contained a single metaphase-arrested nucleus; (**F**,**F’**) In some cases condensed polar body rosettes were visible. (DAPI (grey) and beta-tubulin (green). Scale bars: 50 μm (**E**,**F**) and 5 μm (**E’**,**F’**).

*spindly*[mat67>RNAi] had severe effects on embryogenesis causing a remarkable embryonic lethality (Figure 2A). The well-established function of Spindly for mitosis suggested that *spindly*[mat67>RNAi] should affect the syncytial mitotic divisions in the early embryo explaining the embryonic lethality upon its depletion (Figure 2A). Indeed, the immunostaining of mitotic spindles in early embryos revealed that 83% (*n* = 100) of the *spindly*[mat67>RNAi] embryos showed abnormal syncytial cleavage divisions (Figure 2B–F). The majority of embryos arrested during metaphase of the first 1–3 mitotic divisions (Figure 2C–F). In 40% of the embryos (*n* = 30), only a single metaphase-arrested mitotic spindle was detected within the egg (Figure 2E,E’), whereas only in 13% of the cases a variable number of two to four mitotic spindles were observed. We also detected in many cases (46.6%) characteristic polar body rosettes [40], suggesting that egg activation occurs and meiosis is able to resume in *spindly*[mat67>RNAi] embryos (Figure 2F,F’). We conclude that Spindly is essential for syncytial cleavage division to commence.

### 2.3. Requirement of Spindly for Syncytial Cleavage Divisions

**Figure 3:**
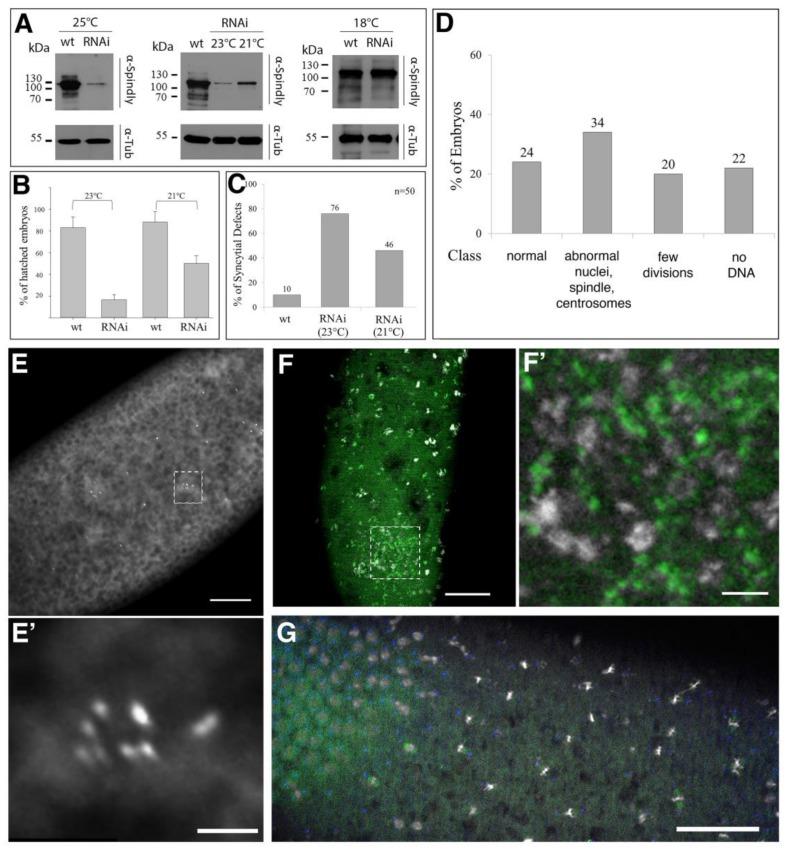
Phenotype of *nos*>>Gal4 driven Spindly knock-down. (**A**) Immunoblot analysis of protein lysates of 0–3 h old *w*^1118^ embryos (wt) or *spindly*[*nos*>RNAi] (RNAi) embryos at different temperatures (loading control—α-Tubulin (Tub); molecular weight standards in kDa); (**B**) Viability of *spindly*[*nos*>RNAi] scored by larval hatching rate (*n* = 100; data represent 3 independent replicates); (**C**) Penetrance of mitotic arrests in syncytial divisions of *spindly*[*nos*>RNAi] embryos featuring phenotypes described in (**D**–**G**); (**D**) Expressivity of syncytial cleavage defects of *spindly*[*nos*>RNAi] embryos at 23 °C. Occurrence of phenotypic classes of *spindly*[*nos*>RNAi] embryos (*n* = 50); (**E**) Syncytial stages with patches of multiple centrosomes (anti-Centrosomin staining); (**E’**) higher magnification of boxed area in (**E**); scale bars: 50 µm (**E**) and 10 µm (**E’**)); (**F**) Condensed chromatin associated with patches of tubulin (detail indicated by yellow box; DAPI (grey), beta-tubulin (green), scale bars represent 50 µm (**F**) and 10 µm (**F’**)); (**G**) Asynchrony of mitotic divisions, as indicated by an area of mitotic divisions and areas enriched in interphase nuclei (scale bar: 50 µm).

The alpha-*tubulin67*>*Gal4* driver exhibits a high level of Gal4 expression in the female germ line, which blocks syncytial cleavages when driving shRNAs directed against *spindly* mRNA. The expression of alpha-*tubulin67*>*Gal4* was first detected when egg chambers leave the germarium (Supplementary Materials Figure S2A). To analyse the requirement of Spindly during syncytial divisions, we expressed *spindly* shRNA using the weaker *nanos (nos)*>Gal4 driver (hereafter *spindly*[*nos*>RNAi]). According to the *nos* expression pattern, nos>Gal4 should be already expressed in the germarium [41,42]. After 3–5 days, *spindly*[*nos*>RNAi] females stopped laying eggs, presumably because the knock-down of Spindly blocked the mitotic division in the germarium, including stem cell divisions. *spindly*[*nos*>RNAi] females produced embryos in which Spindly was efficiently knocked down in a temperature-dependent fashion (Figure 3A–C). At 21 °C to 25 °C, *spindly*[*nos*>RNAi] efficiently depleted Spindly protein in early embryos, being the most efficient at 23 °C to 25 °C (Figure 3A). *spindly*[*nos*>RNAi] embryos exhibited temperature-dependent rates of survival: at 23 °C only 16% (*n* = 100) of embryos hatched, while at 21 °C, about half of the *spindly*[*nos*>RNAi] embryos survived to larval stages (*n* = 100) (Figure 3B). Immunostaining of *spindly*[*nos*>RNAi] embryos was performed to further analyse the cause of the embryonic lethality and revealed a requirement of Spindly for normal syncytial divisions (Figure 3C).

Fluorescence microscopy was used to assess the mitotic defects in *spindly*[*nos*>RNAi] embryos by analysing centrosome positioning and mitotic spindle morphology. At 23 °C, the majority of *spindly*[*nos*>RNAi] embryos had arrested before the end of syncytial divisions (76% *n* = 50; Figure 3C). We further divided the phenotypes of these embryos into different classes (Figure 3D). 22% of all embryos did not show any DNA staining, while 20% arrested development after a few rounds of syncytial mitotic cycles. Of this latter class, 10% of the embryos were characterised by patches of multiple centrosomes (Figure 3E,E’). Another class exhibited the abnormal organisation of the mitotic spindle, where condensed chromatin was found associated with patches of tubulin (12%, *n* = 50; Figure 3F,F’). Unlike wild-type embryos, the nuclei of *spindly*[*nos*>RNAi] embryos were unequally distributed in the cortical cytoplasm of the embryos and the regular spacing of the mitotic domains was lost (Figure 3G). In summary, at the highest temperature tested, *spindly*[*nos*>RNAi] resulted in severe disorganisation of centrosome distribution, chromatin structure, and nuclear distribution in syncytial stages.

**Figure 4:**
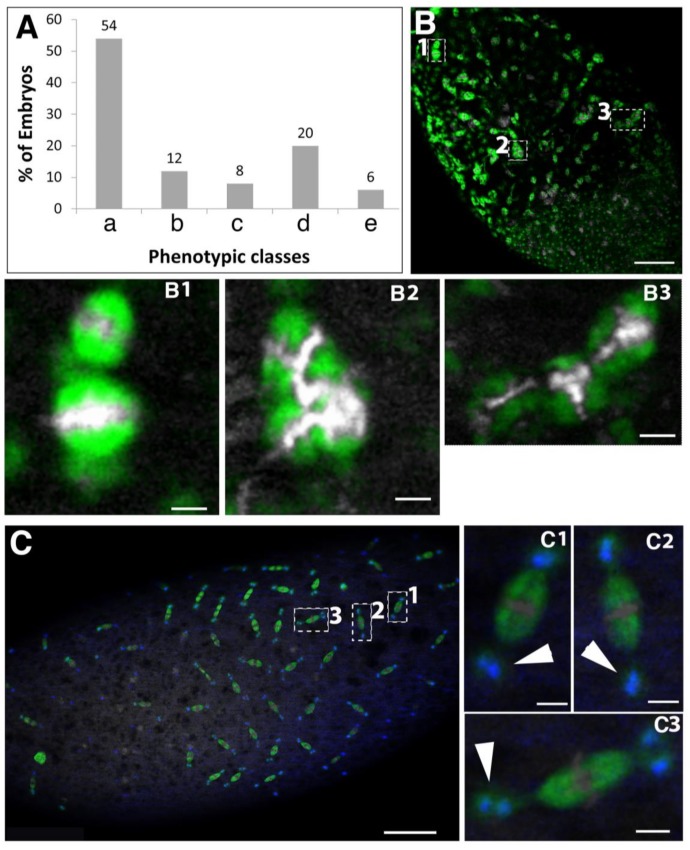

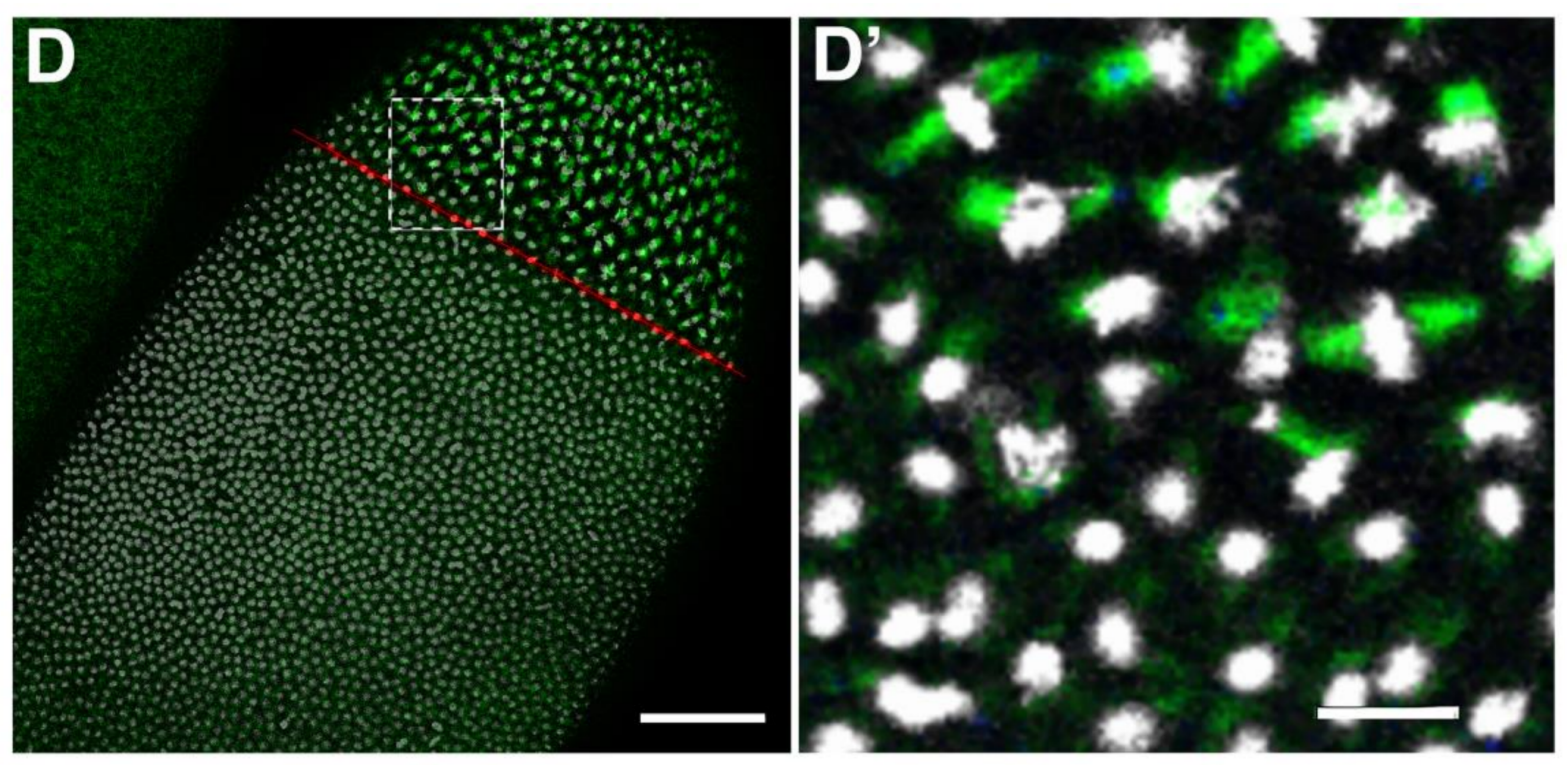
Expressivity of syncytial cleavage defects in *spindly*[*nos*>RNAi] embryos. (**A**) *spindly*[*nos*>RNAi] embryos raised at 21 °C displayed a range of mitotic defects of the following classes (*n* = 50): (a) normal development; (b) abnormal mitotic figures, examples in (**B**); (c) centrosome abnormalities (detachment, monopolar spindles, centrosome clustering; examples in (**C**)); (d) uneven spacing of the mitotic domains and asynchrony of nuclear divisions (examples in (**D**)); (e) dead embryos at later stages; (**B**) Abnormal mitotic figures; irregular spacing between adjacent mitotic domains. Details are shown from the dashed yellow boxes in merged panel. (**B1**) Mitotic spindles collapse and fuse; (**B2**) abnormal tubulin-rich structures associated with chromatin; (**B3**) tri-polar spindle (Scale bars represent 50 μm (**B**) and 5 μm (**B1**–**B3**); (**C**) Centrosome abnormalities: Defects in cohesion between centrosomes and spindle poles. Details from merged panel marked with a dashed yellow box: (**C1**) a pair of centrosomes associated to a single spindle pole (marked by arrowhead); (**C2**,**C3**) centrosomes detach from one (**C2**) or both poles (**C3**); arrowheads point to detaching centrosomes. Scale bar represents 50 μm (**C**) and 5 μm (**C1**–**C3**); (**D**) embryo with domains of distinct mitotic rate; red line marks boundary between a region of interphase nuclei and a region with mitotic divisions. (**D’**) higher magnification of boxed area in (**D**). Scale bars represent 50 μm (**D**) and 10 μm (**D’**).

At 21 °C, only just under 50% of *spindly*[*nos*>RNAi] embryos failed to hatch and exhibited defects in the synchrony of syncytial divisions and various spindle defects (Figure 3C and Figure 4A). The most severe spindle arrests might result from the abnormal spacing of the mitotic domains in the cortex of the syncytial embryo. Often mitotic spindles appeared to be stacked together, fusing into highly abnormal structures with accumulations of tubulin (Figure 4B). About 20% of the abnormal embryos exhibited various centrosome defects, including centrosomes that are detaching from one or both spindle poles or a pair of centrosomes that are associated with one or both poles (Figure 4C). The phenotypes suggest that centrosomes might undergo abnormal replication or they detach and move away from the spindle pole or that free centrosomes are left behind by nuclei dropping out of the cortex. The results suggest that the lower levels of *spindly*[*nos*>RNAi] mediated knock-down produced a threshold of Spindly activity that uncovers its role in spindle assembly and maintenance. 

In early embryos, the observed fusion of mitotic spindles suggests a function of Spindly in the separation of mitotic domains during cortical syncytial divisions. A characteristic feature of *spindly*[*nos*>RNAi] syncytial embryos was a defect in synchrony of syncytial divisions. *spindly*[*nos*>RNAi] contained areas with different rates of mitotic activity, i.e., part of the embryo contained interphase nuclei, while in adjacent areas nuclei were still in mitotic phases (Figure 4D). This asynchrony in mitotic divisions and the uneven spacing of nuclei is quite common in mutants affecting mitotic progression and may suggest that Spindly protein levels are important for the wave-like progression of mitotic divisions in the syncytial embryo [43].

### 2.4. Requirements of Spindly Protein Domains in Early Development

**Figure 5:**
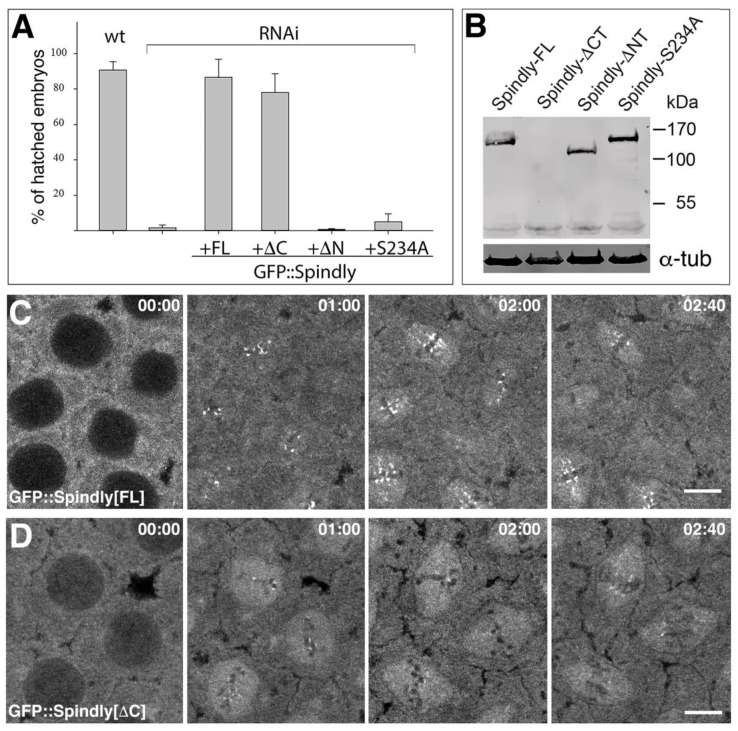

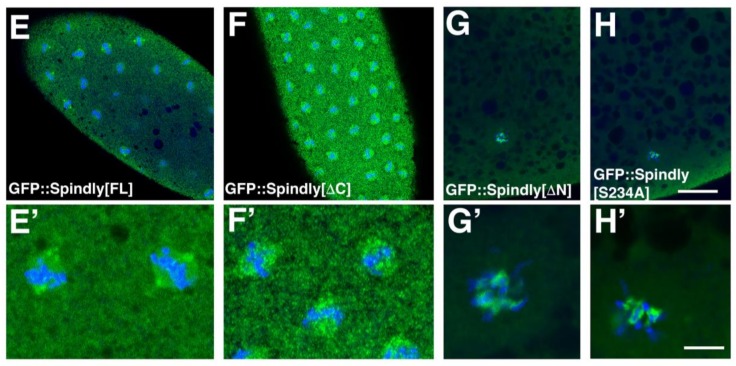
Domain-function analyses of Spindly in the embryo. (**A**) Larval hatching rates in wild type (wt; *w*^1118^), *spindly*[*mat67*>>RNAi] (RNAi) and *spindly*[*mat67*>>RNAi] embryos expressing either GFP::Spindly full-length (+FL), GFP::Spindly^ΔCt^ (+ΔCt), GFP::Spindly^ΔNt^ (+ΔNt) or GFP::Spindly^S234A^ (+S234A) (*n* = 100); (**B**) Western blot analysis of lysates obtained from *spindly*[*mat67*>>RNAi] embryos expressing full length GFP::Spindly (Spindly-FL), GFP::Spindly[Δ-Cterm] (Spindly-ΔC), GFP::Spindly[Δ-Nterm] (Spindly-ΔN), GFP::Spindly[S234A] (Spindly-S234A). Affinity-purified anti Spindly antibodies were used for the analysis, which recognise the C-terminal domain of the protein. Therefore, no signal was detected from the GFP::Spindly[Δ-Cterm] construct; (**C**,**D**) Single frames from time-lapse movies (Supplementary Materials Movies 6,7, respectively) depicting syncytial cleavage division 12 of *spindly*[*mat67*>>RNAi] embryos expressing either full length GFP::Spindly protein (**C**) or GFP::Spindly lacking the C-terminal domain (Spindly-ΔC) (**D**); Note dynamic localisation of GFP::Spindly accumulating in punctate labelling associated with chromosomes, negatively stained darker areas. Scale bars: 5 µm; (**E**–**H**) *spindly*[*mat67*>>RNAi] embryos expressing either GFP::Spindly full-length (GFP::Spindly[FL]) (**E**,**E’**), GFP::Spindly^ΔCt^ (GFP::Spindly[ΔC]) (**F**,**F’**), GFP::Spindly^ΔN^ (GFP::Spindly[ΔN]) (**G,G’**) or GFP::Spindly^S234A^ (GFP::Spindly[S234A], (**H**,**H’**) fixed and stained with anti-GFP (green) antibodies and DAPI (blue); (**E’**–**H’**) show corresponding higher power micrographs. Note accumulation of GFP::Spindly constructs to condensed chromosomes in (**E’**,**F’**); (**G**,**G’**,**H**,**H’**) show polar body rosettes; note the accumulation of GFP::Spindly constructs with the condensed chromosomes. Scale bars represent 50 µm for (**E**–**H**), and 5 µm for (**E’**–**H’**), respectively.

RNAi mediated knock-down of Spindly produced phenotypes that are consistent with a previously known function of Spindly during mitosis. One well-known caveat of RNAi is that associated phenotypes might be produced not (only) by the depletion of the cognate mRNA, but by the depletion of other mRNAs that share sequence similarity. To exclude that the effects of the knock-down were caused by such off-target effects we generated an RNAi-resistant construct encoding an N-terminally GFP-tagged full-length version of Spindly, in which the shRNA target site was mutated without changing the polypeptide sequence. The overexpression of the RNAi-resistant GFP-tagged Spindly transgene restored the viability of *spindly*[mat67>RNAi] embryos to wild-type levels (Figure 5A). The rescue also provided us with a basis for investigating the requirements for different protein domains of Spindly for its function and localisation (see Figure 1A). We found that the C-terminal region was largely dispensable for the rescue of *spindly*[mat67>RNAi] embryos, while the N-terminal region and the highly conserved Serine 234 residue within the Spindly box were essential for the Spindly function (Figure 5A). As a control for the expression of the protein constructs in the *spindly*[mat67>RNAi] background, we analysed the protein levels by Western blotting (Figure 5B). The GFP::Spindly[S234A] and the GFP::Spindly[Δ-N-term] proteins were expressed at levels that are similar to the GFP-tagged full length protein, indicating that the lack of rescue was not caused by poor protein stability or expression.

Our results indicated that the C-terminal half of Spindly was dispensable for its function in the embryo. However, in neuroblasts, the C-terminal region was required for the localisation of the protein to kinetochore (Figure 1C). This discrepancy raised the possibilities that either Spindly has kinetochore-independent functions that are sufficient to complement its loss in the embryo, or that Spindly binds to the kinetochore in a cell-type specific manner. To discriminate between these possibilities, we imaged GFP::Spindly and the GFP::Spindly[Δ-C-term] protein in *spindly*[mat67>RNAi] embryos (Figure 5C,D). We found that the GFP::Spindly accumulated on punctate structures as consistent with a localisation to the kinetochore in a dynamic fashion (Figure 5C,E; Supplementary Materials, Movie 6). In prometaphase, GFP::Spindly associated in a dot-like fashion with areas of condensed chromatin and during metaphase these punctae were lined up in a series of dots at the metaphase plate (Figure 5C). As anaphase proceeded, the punctae moved towards the spindle poles and eventually accumulated at one side of the reforming nucleus (Supplementary Materials Movie 6). The GFP::Spindly[Δ-C-term] protein behaved similarly to the full-length GFP::Spindly protein, however the accumulation into punctae on the condensed chromosomes were less pronounced compared to the full-length protein (Figure 5D,F). Nevertheless, the dynamics of the GFP::Spindly[Δ-C-term] punctae during mitosis was comparable to the full-length protein (Supplementary Materials, Movie 7). We conclude that, despite the requirement of the C-terminal region in neuroblasts, Spindly is able to localise without the C-terminal region in a similar fashion to the full-length protein, albeit in a somewhat reduced level.

We also examined the distribution of the GFP::Spindly[S234A] and the GFP::Spindly[Δ-N-term] proteins, which were not able to rescue the *spindly*[mat67>RNAi] lethality (Figure 5G,H). The phenotype of such embryos was largely equal to the phenotype that was described for *spindly*[mat67>RNAi] (Figure 2), with most embryos exhibiting arrested meiosis stages with polar body rosettes (Figure 5G’,H’). Interestingly, the mutant GFP::Spindly proteins were closely associated with the condensed chromosomes of the rosettes, suggesting that, similar to the neuroblasts, their chromosome binding was unimpaired, but that these proteins are otherwise non-functional. Furthermore, the result with the C-terminally deleted proteins demonstrated that *Drosophila* Spindly exhibits a differential cell-type specific requirement for its C-terminal region for binding to the kinetochore.

### 2.5. Spindly Overexpression Causes Defects in Egg Shape and Embryogenesis

**Figure 6:**
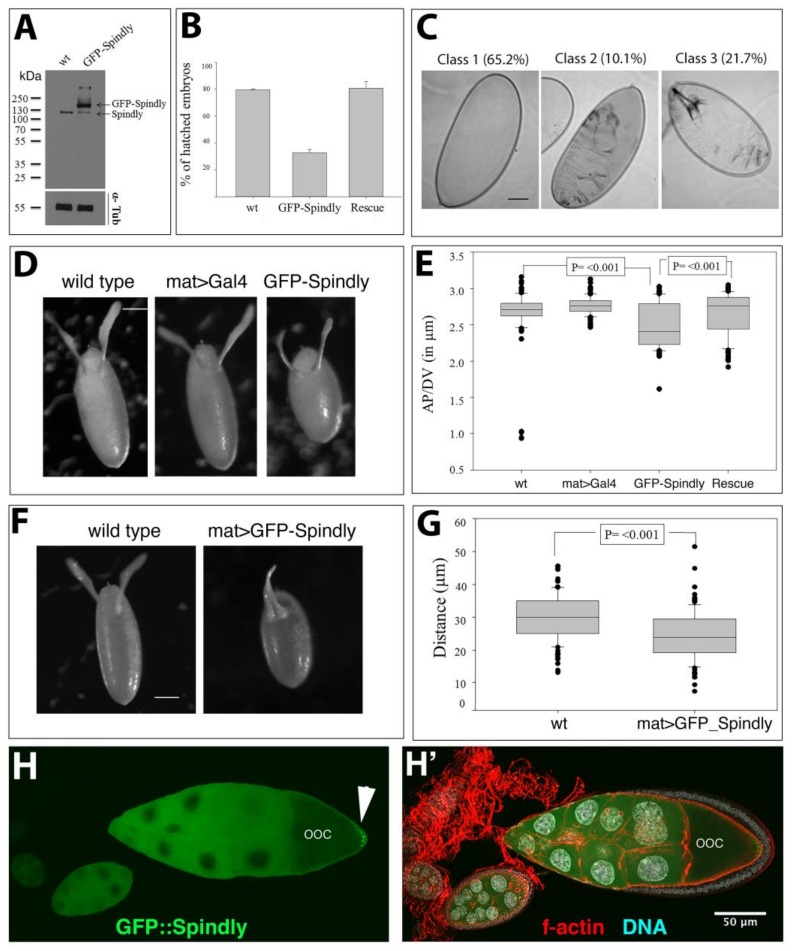
Overexpression of Spindly affects oogenesis and embryogenesis. (**A**) Immunoblot analysis of Spindly in lysates from wild-type (*wt*) and *nos>>Gal4*::*UAS>>GFP::Spindly* (GFP::Spindly) embryos. Endogenous Spindly and GFP::Spindly bands are indicated (α-tubulin—loading control; molecular weight markers in kDa); (**B**) Survival rate of embryos upon Spindly overexpression scored by larval hatching (*n* = 100). Viability was restored by knock-down of endogenous Spindly with UAS>>Spindly[RNAi] (Rescue); (**C**) Larval cuticle defects in *mat67>>Gal4*:*UAS>>GFP::Spindly.* The majority of embryos died before cuticle deposition (65.2%, class 1). The remaining 31.8% of embryos exhibited dorsal holes (class 2) or segmentation defects (class 3) (*n* = 138); (**D**) Eggs laid by *mat67>>Gal4*::*UAS>>GFP::Spindly* females exhibited abnormal rounded shape (48%, *n* = 100). Scale bar represents 50 μm; (**E**) Quantification of egg size by calculating the ratio between the length (A/P axis) and the width (D/V axis) for a total of 100 eggs (Mann-Whitney Test (*p* ≤ 0.001). This phenotype is suppressed by UAS>>Spindly[RNAi] knock-down of endogenous Spindly (Rescue); (**F**) Moderate ventralisation defect in chorions of *mat67>>Gal4*::*UAS>>GFP::Spindly* eggs. The dorsal appendages were shorter and apposed closer to each other along the dorsal midline compared to controls. Scale bar represents 50 μm; (**G**) Quantification of the distance between dorsal appendages shown in (**F**) (Mann-Whitney Test *p* ≤ 0.001; *n* = 100); (**H**) Part of an ovariole from an ovary of a *mat67>>Gal4*::*UAS>>GFP::Spindly* female fixed and stained with antibodies against GFP (green), f-actin (red) and DAPI (blue). Note the accumulation of GFP::Spindly (arrowhead) at the posterior pole of the oocyte (ooc) in the egg chamber (stage 10). Scale bar: 50 µm.

The loss-of-function studies reported here demonstrated that maternally supplied Spindly is essential for sustaining the syncytial cell cycles in the embryo. Importantly, the phenotypes were specific for Spindly RNAi knockdown, as a full rescue of the defects was observed by the expression of an RNAi-resistant Spindly protein. In the course of these experiments, we noted that the expression of GFP::Spindly using the maternal *alpha-tubulin67c>Gal4,* in a wild-type background that is caused defects in embryogenesis. In *mat67>>GFP::Spindly* embryos, levels of Spindly-GFP were higher than endogenous protein levels in lysates from 0–3 h-old embryos at 25 °C (Figure 6A).

The majority (70%, *n* = 100) of *mat67>>GFP*::*Spindly* embryos did not hatch, but the co-expression of Spindly[shRNA] was able to fully rescue this effect, indicating that this phenotype was caused by an altered level of Spindly protein, rather than by an abnormal effect of the GFP::Spindly fusion protein (Figure 6B). The embryonic lethality of *mat67>>GFP::Spindly* resulted in three classes of larval cuticle phenotypes (Figure 6C). Within the largest class, embryos did not form any cuticle, suggesting that Spindly is either involved in cuticle formation or the embryos died early in development. The remaining embryos exhibited cuticle phenotypes, suggesting a range of morphogenetic defects (Figure 6C). In addition to embryonic defects, the normal egg shape was impaired by *mat67>>GFP::Spindly* expression, resulting in eggs that were shorter along their anterior-posterior axis (Figure 6D,E). In addition, the morphology of the dorsal-anterior appendages of the chorion suggested a mild ventralisation phenotype as the distance between the attachments of the appendages was significantly shorter in *mat67>>GFP::Spindly* eggs (Figure 6F,G). These data indicate that the overexpression of Spindly in the female germ line affects egg formation and patterning in oogenesis and embryonic survival.

To shed some light on the way how Spindly overexpression affected oogenesis, we examined the distribution of GFP::Spindly in the egg chambers (Figure 6H,H’). We find that in stage 10, egg chambers the overexpressed GFP::Spindly in the germ line is equally distributed in the nurse cells, but in the oocyte the protein accumulates in the posterior pole of the oocyte cortex. The distribution of the GFP::Spindly protein was similar in *spindly*[mat67>RNAi] ovaries, indicating that the excess overexpressed protein localised like the endogenous protein (Supplementary Materials Figure S2B). The localisation of Spindly protein in stage 10 egg chambers is reminiscent of the localisation of components that are transported along microtubules towards their plus ends, which become localised to the posterior cortex of the oocyte. These results are consistent with the localisation of overexpressed Spindly protein in cultured cells [9], and suggest that the overexpressed GFP::Spindly protein might interfere with the function of microtubule plus ends or proteins that are associated with these.

### 2.6. Requirement of Spindly in Ovarian Border Cell Migration

Knockdown of Spindly drastically alters S2 cell morphology in interphase: Spindly-depleted cells have malformed actin lamellae and abnormal microtubules bundles that protrude from the cell body [9]. Furthermore, the overexpression of a GFP-tagged Spindly revealed its accumulation at the plus ends of microtubules in S2 cells and in the oocyte (Figure 6H; [9]). We therefore addressed whether Spindly has a role in regulating the motile behaviour of cells.

**Figure 7:**
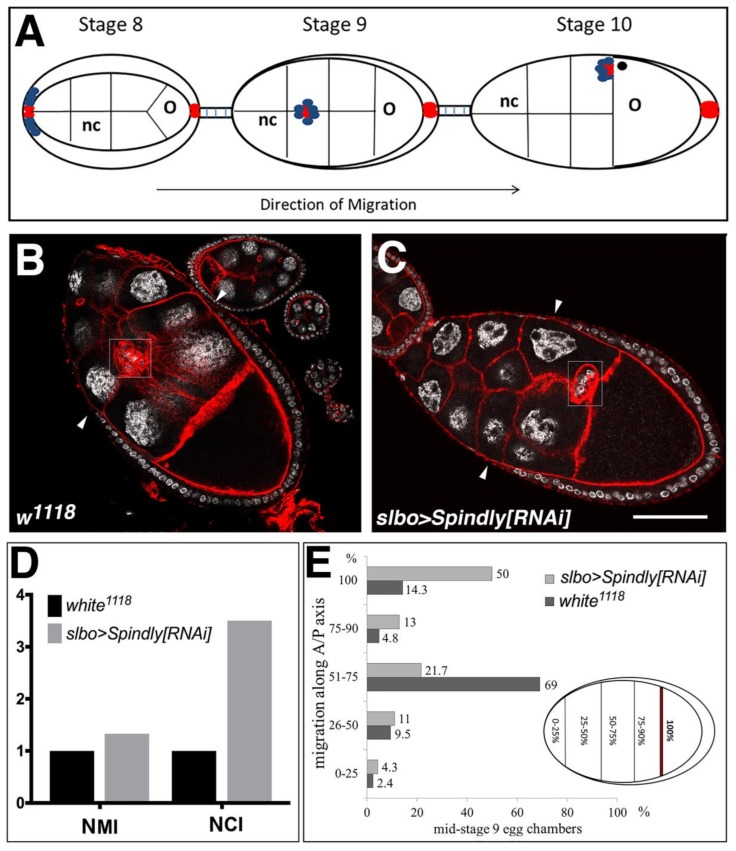
Spindly knockdown in migrating ovarian border cells. (**A**) Schematic drawing of border cell migration and outer follicle cells rearrangement. Egg chambers are depicted at stage 8–10 of oogenesis, where each egg chamber consists of 16 germ line cells (15 nurse cells (nc) and one oocyte (o)) and are surrounded by a layer of somatic follicle cells. At stage 8, polar cells (in red) at the anterior of the follicular epithelium recruit neighbouring cells (in blue) to form the migrating border cell cluster. At stage 9, the cluster delaminates from the epithelium and starts migrating posteriorly towards the oocyte and arriving at the nurse-cell-oocyte boundary at stage 10; (**B**,**C**) Immunostaining of stage 9 egg chambers from wild-type (*w*^1118^) (**B**) and *slbo>Gal4;UAS>SpindlyRNAi* females (**C**); (DAPI (grey), f-actin (red). Arrowheads indicate the border between squamous and columnar follicle epithelium; scale bar: 50 μm; (**D**) Normalised migration index (NMI) and Normalised completion index (NCI) are indicated; for calculation see Materials and Methods; (**E**) Quantification of the phenotype described in (**B**,**C**). Premature border cell migration was observed in 50% of stage 9 egg-chambers (*n* = 42).

The border cells (BCs) in the *Drosophila* ovary provide an excellent model system to study genetic requirements of collective cell migration [44] The BCs form a cluster of 6–10 post-mitotic cells at the anterior domain of somatic follicular epithelium of the egg chamber around stage 8 of egg chamber development (Figure 7A). Subsequently, BCs undergo an epithelial-mesenchymal transition and migrate posteriorly as a coherent cluster, reaching the nurse cell-oocyte boundary by stage 10 of egg-chamber development (Figure 7A) [44]. Spindly expression was knocked down by driving shRNAi with the BC-specific *slow border cells* (*slbo*) Gal4 driver (*slbo>Gal4*) [45]. In wild-type egg chambers the majority of BC clusters had migrated halfway through the egg chamber at late stage 9 (69%, *n* = 42), while a small percentage (14.3%) had already completed their migration at this stage of egg-chamber development (Figure 7B,D,E). In ovaries from *slbo*>Spindly[RNAi] females, 50% (*n* = 46) of the BC clusters had completed migration at mid-stage 9 of oogenesis (Figure 7C–E). To compare the BC cluster migratory phenotypes upon Spindly-RNAi in a quantitative fashion, the normalised migration (MI) and the completion (CI) indices were calculated [46]. Values of both indices suggested that the down-regulation of Spindly protein level confers a migratory advantage to BC (Figure 7D). Thus, we concluded that knockdown of Spindly caused the BC cluster to migrate either earlier or faster along the anterior-posterior axis towards the oocyte, suggesting that Spindly negatively regulates border cell migration.

**Figure 8:**
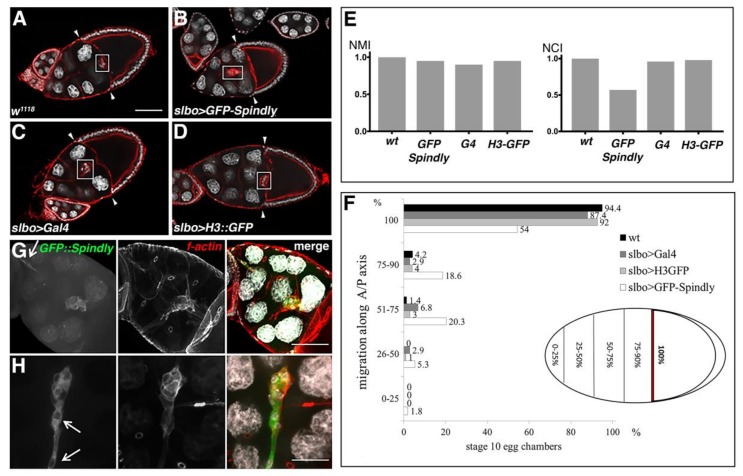
Spindly overexpression in migrating ovarian border cells. (**A**–**D**) Confocal optical sections of fixed and immunolabeled egg-chambers at stage 10 of oogenesis of the indicated genotypes. Migration of border cells was delayed by GFP::Spindly overexpression (**B**) compared to *w*^1118^ (**A**), *slbo>Gal4* (**C**) and *slbo>Gal4;H3::GFP* (**D**) (*slbo>H3::GFP*) as controls. Arrowheads indicate the border between squamous and columnar follicle epithelium DAPI (grey); f-actin (red); scale bars: 50 μm; (**E**) Comparison of normalised migration indices (NMI) and normalised completion index (NCI); (**F**) Quantification of the phenotype described in (**E**). 46% of the clusters overexpressing Spindly did not complete migration by stage 10 of egg-chamber development (*n* = 113); (**G**) Morphology of border cell clusters in egg chambers of *slbo>Gal4; UAS>GFP::Spindly* females. Confocal optical section of stage 10 egg-chambers (maximum-intensity projection of 10 μm z-stack at a spacing of 1 μm). Loss of border cell cluster integrity is marked by white arrows. In 11,5% of cases the border cell cluster had lost integrity or in most severe cases single cells had detached from the main cluster (**H**) and migration was delayed (*n* = 113) (DAPI (grey), f-actin (red), GFP (green); scale bars represent 20 μm).

The sensitivity of BCs to Spindly levels prompted us to test the effect of Spindly overexpression on their migration. Specifically, we asked whether overexpression of Spindly could change BC migration at stage 10 of egg chamber development (Figure 8E,F). Indeed, when analysing the migration of the border cells, we found that in 46% (*n* = 113) of BC clusters their migration towards the oocyte was delayed at stage 10 of egg-chamber development (Figure 8A,B,E,F). While *slbo*>Gal4 females on their own also exhibited a mild incomplete migration phenotype at stage 10 (12.6%; *n* = 103), the penetrance of the phenotype was significantly lower when compared to *slbo>>GFP::Spindly* overexpression (Figure 8C,E,F). As additional control, the overexpression of GFP-tagged histone H3 did not alter border cell migration (*n* = 100), supporting the result that the increased Spindly expression causes incomplete border cell migration (Figure 8D–F). When comparing the normalised migration and completion indices, overexpression of GFP::Spindly reduced the completion of BC migration to about 50% (Figure 8E). We conclude that the overexpression of GFP::Spindly produces the opposite phenotype as the knockdown of Spindly supporting our conclusion that Spindly is involved in timely migration of the BC in oogenesis.

Overexpression of Spindly in BCs also affected their morphology in a way suggesting abnormal adhesive properties and cytoskeletal architecture. Many of the BC clusters lost their integrity and the cell-cell contacts between the BCs were compromised to the extent that individual cells detached from the BC cluster, and thus were apparently migrating as individual cells (9.7%, *n* = 113) (Figure 8G,H). These results suggested that excess Spindly negatively affects the migration and the integrity of the border cell cluster during migration. We conclude that correct Spindly levels are required in post-mitotic follicle BC to control or modulate their migration possibly by controlling the integrity and adhesion of the cluster.

## 3. Discussion

### 3.1. Spindly Function in Early Drosophila Embryogenesis

In this work, we present the first investigation of the mitotic and post-mitotic activities of *Drosophila* Spindly in a variety of cellular and developmental processes. The temperature sensitive Gal4/UAS system in combination with shRNA-mediated knockdown provided a means to generate embryos with different maternally derived levels of Spindly protein; these combinations produced phenotypes as expected from a series of hypomorphic to amorphic mutant alleles. Consistent with its central role in the mitotic checkpoint, *spindly*[RNAi] embryos exhibit mild to severe defects in mitosis. We showed that Spindly has an important role in the transition from a mature oocyte into cleavage stages of embryonic development. Analysis of nuclear proliferation in *spindly*[RNAi] embryos revealed abnormal mitotic progression, which results in an early arrest of embryo development.

The phenotype of Spindly[RNAi] embryos in mitosis are reminiscent of defects that were observed in *Drosophila* embryos injected with Dynein inhibitors [47]. It is well known that the maintenance of the spacing and architecture of the spindle machinery is achieved by the coordinated and complementary activity of different spindle-associated Dynein and Kinesin motor proteins [48,49]. Loss of Spindly function might alter the balance between the opposing forces that govern spindle stability, resulting in defective spindle morphology and chromosome segregation. In *Drosophila*, Dynein plays a major role in maintaining a tight association between the centrosomes and the spindle poles [50,51]. Therefore, in Spindly[RNAi] embryos, the replication of detached centrosomes and the association of these free centrosomes to neighbouring spindles likely causes further aberrant mitoses. Although a direct interaction between Spindly and Dynein in *Drosophila* has yet to be demonstrated, our results support the idea that the knock-down of Spindly reflects a loss of Dynein function in establishing and maintaining stable kinetochore-microtubule interactions [27].

Human Spindly forms a ternary complex with Dynein and Dynactin in order to fulfil its functions [9]. The first coiled-coiled domain in the N-terminal portion of Spindly is responsible for the interaction with Dynein LIC-1 [52]. The Spindly box contains highly conserved residues, S256, F258 in human, and F199 in *C. elegans*, which are essential for the interaction with the Dynactin complex, and for the binding of dynein to kinetochores [29,34,52]. In our study, we tested the requirement of these domains for the mitotic function of Spindly. We found that Spindly protein constructs lacking the N-terminal domain or containing a mutation in the conserved S234 residue (S256 in human Spindly) are still able to localise to kinetochores in neuroblasts, but were unable to rescue the lethality that was caused by the knockdown of maternal Spindly in the embryo. We conclude that the requirement of the N-terminal half and the Spindly box for the interaction of Spindly with Dynein may be evolutionarily conserved.

The C-terminal portion of human Spindly is crucial for the localisation of the protein at kinetochores [30]. Here, we show for the first time that in *Drosophila* the C-terminal region exhibits a cell-type specific requirement for kinetochore binding. The C-terminal domain is essential for kinetochore binding in larval neuroblasts, but it is dispensable for kinetochore binding in the early embryo. In the embryo, Spindly protein lacking the C-terminal domain is still functional as it is able to rescue the lethality of the RNAi knockdown of Spindly. Other protein domains are likely to contribute to Spindly localisation making the C-terminal region no longer essential for kinetochore binding in early embryos. One possibility would be that the composition of the outer kinetochore differs between the early embryos and larval neuroblasts, and therefore, Spindly may achieve kinetochore binding by interacting with different binding partners. Similar cell-type specific differences between neuroblasts and syncytial embryos have been reported for the assembly of the kinetochore component *rough deal* (*rod*) [53]. A mutant form of Rod, Rod^Z3^, was unable to localise to kinetochores in syncytial embryos, but is localised, albeit in a reduced fashion, to kinetochores in larval neuroblasts. Therefore, it is likely that there is a profound difference for the requirement of stable kinetochore-microtubule interactions in syncytial vs. post-embryonic cell divisions. This difference may also be reflected by the robustness of mitotic divisions in *Drosophila* upon inactivation of the SAC. The important SAC component Mad2 is dispensable for viability and fertility in the fly, while other components, e.g., the RZZ complex, are essential for the progression through mitosis [14,53]. Our study adds Spindly to the latter group of SAC components and indicates that like the RZZ complex Spindly might have SAC independent functions that are essential for the progression through mitosis in *Drosophila* embryos.

### 3.2. Spindly Overexpression Affects Dynein-Dependent Processes in Egg Patterning

Spindly overexpression led to embryonic lethality, altered egg morphology and a mild ventralisation phenotype. Within the ovary, spherical egg chambers elongate dramatically until they acquire the typical ellipsoid shape of the mature egg. The contribution of the somatic follicular epithelium to the process has been well characterised. Egg elongation relies on the interaction between a polarised actin network in the follicle cells and a polarised arrangement of the basal membrane components [54,55,56]. Maternal effect mutations also often result in a round egg phenotype [57]. One of the best studied class is the so-called dumpless phenotype. This phenotype is caused by reduced cortical, radial, and/or ring canal associated actin bundles or defects in the ring canal morphogenesis required for the fast transport of maternal products from the nurse cells into the oocyte [58]. The slow transport of nurse cell products into the oocyte transport is Dynein-based [59] and mutations in the Dynein light intermediate chain also cause a round egg phenotype and ventralisation (Hüttinger and Müller, unpublished data). We therefore propose that Spindly overexpression might interfere with Dynein-based transport, resulting in altered egg morphology.

The egg shape defect upon Spindly overexpression was associated with a mild ventralisation phenotype. The establishment of dorsal/ventral polarity occurs during mid-oogenesis and ultimately relies upon a dorsalising signal that is generated by the *gurken* gene [60,61]. Transport of *gurken* mRNA to an anterior-dorsal position is dependent on Dynein and requires anchoring by microtubules surrounding the oocyte nucleus [62,63,64]. Defects in Dynein and its co-factors cause the displacement of the oocyte nucleus, and consequently, of Gurken away from its anterior-dorsal position, resulting in the ventralisation of the eggs [59,65,66,67,68]. Since we did not observe any egg shape and ventralisation defects in eggs derived from mat67>Spindly RNAi females, it is unlikely that Spindly acts in these processes. We therefore conclude that Spindly interferes with Dynein dependent process by either directly binding to Dynein itself or another of its cofactors, thereby compromising their function. Loss of the Dynein cofactor Asunder in the germ line results in a similar egg shape and ventralisation defects, as we observed upon the overexpression of Spindly, and therefore represents one candidate target affected by Spindly overexpression [67]. The localization of the excess Spindly protein at the posterior pole of the oocyte also suggests that the protein interferes with Dynein function as Dhc and Dlic have also been reported to accumulate in this area [10,67].

### 3.3. A Role for Spindly in Cell Migration

The loss- and gain-of-function experiments performed on follicular border cells have opened an ideal system to test the role of Spindly in the regulation of cell migration in vivo. We found that the downregulation of Spindly positively regulates the migration of border cells towards the oocyte. Conversely overexpression of GFP-tagged Spindly resulted in an incomplete migration phenotype and abnormal cluster morphology. The Dynein light chains, Lis-1, and Nud-E have been implicated in border cell migration; however, in contrast to Spindly-RNAi, knock-down of these proteins delayed border cell migration [69]. In addition, Dynein chains are required for the normal organisation of the cluster, proper localisation of adhesion molecules and epithelial cell polarity [69,70]. These data suggest that altering the activity of dynein might cause defects in border cell cluster morphology. We hypothesise that Spindly gain-of-function might have similar consequences to the overexpression of Dynamitin [66,71,72], resulting in the inhibition of dynein activity and altered distribution of polarity markers and/or adhesion molecules. This interpretation would fit with the mild-ventralisation of the egg upon Spindly overexpression, a phenotype that could arise from reduced Dynein activity, and therefore the detachment of the oocyte nucleus from the anterior-dorsal cortex.

## 4. Materials and Methods

### 4.1. Drosophila Strains and Culture

Flies were kept on standard medium and grown accordingly to standard laboratory procedures. *white*^1118^ serves as wild-type control strain (Bloomington Stock Centre, Bloomington, IN, USA). To express genes ectopically and for the RNAi experiments, the Gal4-UAS system was used [37]. The Gal4 lines used were: *nanos*>Gal4 VP16 (Ulrike Gaul), *maternal tubulin 67c*>Gal4, *slbo*>Gal4 (Denise Montell). The UAS lines used were: p*UASp*>GFP::Spindly constructs (this work), *UAS*p>Spindly RNAi (FBst0034933, 34933 *Bloomington Stock Centre*), *UAS*>*H*_3_-*GFP*, *worniu*-GAL4 [73], and UAS-mCherry::tubulin [74]. Transgenic flies harbouring the p*UASp*>GFP::Spindly constructs were generated by PhiC31–mediated sequence-directed transformation using an attP site at the genomic location 68A4.

### 4.2. Embryo Collection

Embryos were collected on yeasted apple juice agar plates. Gal4>Spindly^RNAi^ females were crossed to homozygous UAS::Spindly^RNAi^ males at indicated temperatures. To determine the hatching rates, embryos from an overnight collection were counted and aged at 25 °C, 23 °C, 21 °C, 18 °C for 48 h. The number of hatched embryos was determined by counting the number of intact, unhatched embryos, and subtracting this value from the total number of embryos collected. For larval cuticle preparation, terminally differentiated embryos were collected and dechorionated in bleach. After extensive washes in PBS-0.1% Triton X-100, embryos were mounted in Hoyer’s mounting solution/lactic acid (1:1). Samples were incubated overnight at 65 °C. Imaging was performed on an Olympus BX-61, TRF microscope system.

### 4.3. Culture and Live Imaging of Larval Neuroblasts and Embryos

Live imaging of isolated neuroblasts was performed using published methods [38]. Brains were dissected from third instar larvae and then incubated in collagenase for 20 min. Brains were then manually dissociated with needles in fibrinogen (Sigma-Aldrich F-3879, Gillingham, UK), and dissolved in Schneider’s medium (SLS-04-351Q) on a 25 mm glass bottom dish (WPI). Fibrinogen was then clotted by the addition of Thrombin (Sigma T7513). Schneider’s medium was then added and a 5 µM slice at the centre of the cell was imaged every 2 min. Imaging was performed using a 100× objective (Oil, NA1.45) on a spinning disk confocal microscope. For embryo live imaging, embryos were dechorionated and mounted on a petri dish (fluorodish, WPI) under halocarbon oil 27 (Sigma). Images were taken at 20 s intervals on a Zeiss LSM880 Airyscan confocal microscope using parallel oversampling with the airyscan module. All data was processed using FIJI [75].

### 4.4. Site-Directed Mutagenesis

An RNAi-resistant GFP-tagged Spindly transgene was generated by mutagenesis PCR targeting the shRNA-binding site (1611-caggacgcggttgatatcaaa-1632) at every third position of each codon, leaving the polypeptide sequence codified unaltered. The shRNA-resistant nucleotide sequence originated was 5’-caagatgccgtggacattaag-3’. A GFP-tagged variant of the full-length *spindly* cDNA cloned in a Gateway vector was kindly provided by Dr E. Griffis (School of Life Sciences, University of Dundee). This construct was used as a template in a site-directed mutagenesis PCR using the following primers (Table 1) for mutagenesis:

The DpnI-digested PCR products were used to transform *E. coli* and positive clones were sequenced to verify that only the appropriate mutation was present. The cDNA codifying for the RNAi-resistant GFP::Spindly was then subcloned in a pUASp K10 vector using KpnI-BamHI sites and was used to generate transgenic animals. The same plasmid vector was used to generate Spindly mutant variants. Primers are available upon request.

### 4.5. Protein Extraction and Western Blotting

0–3 h-old embryos were collected on apple juice plates at different temperatures, as indicated in the text. After dechorionisation, embryos were lysed in RIPA Buffer (50 mM Tris-HCl pH 8.0, 150 mM NaCl, 1% NP-40, 0.5% Sodiumdeoxycholate, 0.1% SDS) containing a mixture of protease inhibitors (Mini-cOmplete EDTA-free-Roche, 1 tablet/10 mL). The homogenised tissue was incubated on ice for 30 min. Subsequently, the samples were centrifuged twice at 13,000 rpm for 30 min at 4 °C to remove insoluble debris. Protein concentration was estimated by reading the absorbance at 280 nm using a photometer (Biophotometer, Eppendorf, Hamburg, Germany). For Western Blotting, 10 μg of proteins for each sample were heat-denatured, resolved on SDS-PAGE, and transferred to PVDF membrane (Whatman, GE Healthcare, Little Chalfont, UK) for immunoblotting. The membrane was immunoblotted with the following primary antibodies: rabbit-anti-Spindly 1:2000 [9], rabbit-anti-actin 1:3000 (Sigma-Aldrich A-2066, Gillingham, UK), and mouse-anti-tubulin 1:2000 (Developmental Studies Hybridoma Bank (DSHB), 12G10; Iowa City, IA, USA). After extensive washes, membrane was incubated with HRP-conjugated antibodies: anti-rabbit-HRP 1:5000 (Thermo Scientific, #31460, Loughborough, UK) and anti-mouse-HRP 1:2000 (Roche, Burgess Hill, UK). Signals were detected using chemi luminescence on X-ray film.

### 4.6. Embryo Immunostaining

Embryos for immunostaining were collected every three hours at different temperatures, as indicated and dechorionated. Embryos were transferred in a 1:1 solution of heptane and 4% formaldehyde in PBS for 20 min. Alternatively, embryos were incubated in a 1:1 solution of PEM Buffer containing 1 μM Taxol (Tocris Bioscience, Bristol, UK) and heptane to which 1 mL of 20% formaldehyde was added for 10 min. Taxol was added to stabilise microtubules and improve the staining of this cytoskeletal component [76]. After fixation, formaldehyde was replaced with methanol and vigorously shaken. After two washes in methanol, embryos were washed in PBS-0.1% Tween-20 (3 times, 15 min each) and incubated in blocking solution for 1 h at room temperature. Primary antibodies were incubated overnight at 4 °C, rotating. Primary antibodies were used at the following dilutions: rabbit anti-Centrosomin 1:250 (kindly provided by Professor J. Raff, Oxford, UK), and mouse anti-β-tubulin 1:50 (DSHB, E7; Iowa City, IW, USA). Fluorophore-conjugated secondary antibodies (Invitrogen by Thermo Fisher Scientific, Paisley, UK) were used at 1:250 and DAPI at 1 µg/mL. Specimens were mounted in MOWIOL/DABCO and imaged on Olympus BX-61, TRF fluorescence microscope. Images were processed using Volocity (Improvision, Perkin Elmer, Seer Green, UK) and Adobe Photoshop.

### 4.7. Ovary Dissection and Immunolabeling

1–3 old mated flies were placed on standard medium containing dry yeast for 24 h at 25 °C prior to dissection. Ovaries were dissected in phosphate buffer (PBS) and were fixed in cold 4% formaldehyde in PBS for 15 min. Ovaries were washed repetitively in PBS- 0.1% Tween-20 and rotated for 2 h in a solution of PBS- 0.1% Triton X-100 supplemented with 5% Foetal Calf Serum (FCS). F-actin staining was performed by adding fluorochrome–conjugated Phalloidin (1:50) (Phalloidin-Alexa594, Invitrogen #A12381). Subsequently, the samples were washed three times in PBS-0.1% Tween-20, 15 min each time on a rocking platform. Nuclei were stained with DAPI (1:1000) for 10 min. Ovaries were embedded in Mowiol/DABCO (Sigma Aldrich) and mounted on coverslips. Microscopy was performed using confocal laser-scanning microscopes (Leica SP2, Milton Keynes, UK), Leica TCS SP8 and Zeiss LSM880, Cambridge, UK). Images were processed using Volocity (Improvision, Perkin Elmer, Seer Green, UK) and Adobe Photoshop. Image J [77] was used for image analysis.

### 4.8. Egg Chamber Staging

Precise identification of egg-chamber stages was based on egg chamber morphology and stage-specific markers. At the transition between stage 8 and 9 of egg-chamber development, concomitantly to the initiation of border cell cluster migration, the anterior follicle cells start to migrate away from each other in an anterior to posterior direction to cover the oocyte. To unequivocally identify mid-stage 9 egg chambers, we took advantage of the morphogenetic rearrangements of the follicular epithelium. Particularly, a line was traced to connect the dorsal and ventral anterior-most cells in the follicular epithelium, and the distance between the position of the follicle cells and the nurse cells (NC)-oocyte boundary was measured. For mid-stage 9 egg chambers, an average distance value of 46 μm was calculated. Image analysis and distance measurements were performed on Image J.

### 4.9. Quantification of the Migration Phenotypes

For scoring the migration of the border cell cluster, the egg chamber was divided in five regions: 0–25% f migration, 26–50% of migration, 51–75% of migration, 75–90% of migration, and 100% complete migration. The distance travelled by the cluster was measured as the distance between the posterior-most follicle cells and the leading border cell. This value was then normalised to the total distance, that is the distance between the posterior-most follicle cells and the NC-oocyte boundary and expressed as percentage. On the base of these measurements, the number of clusters falling in each region was counted and results represented in histograms. Image analysis and distance measurements were performed on Image J. Migration (MI) and Completion (CI) indices were calculated, as previously described [46]. The MI is the measure of the mean distance that is travelled by the cluster. This index was calculated with the following formula:

$$\mathrm{MI}=\frac{1\times n\;\left(100\%\right)+0.75\times n\;\left(75\%\right)+0.5\times n\;\left(50\%\right)+0.25\times n\left(25\%\right)+0\times n\left(0\%\right)+0.5\times n\left(\mathrm{splitcluster}\right)}{N\;\left(\mathrm{total}\right)}$$

In the formula, n (100%) represents the number of clusters that have completed migration, reaching the NC-oocyte border, n (75%), n (50%), and n (25%) represent the number of clusters that have migrated 75%, 50% and 25% of the total distance, respectively. The formula includes also the number of dissociated clusters (n splitcluster). The CI index represents the number of clusters that have completed migration. CI is calculated as the ratio between the number of clusters that have migrated the total distance [n(100%)] and the total number of egg chambers examined [n(total)].

$$\mathrm{CI}=\frac{n\;\left(100\%\right)}{n\;\left(\mathrm{total}\right)}$$

The indices were normalised to the wild-type control.

## Figures and Tables

**Table 1:** Primer.

Primer	5′➔3′
Forward	caagatgccgtggacattaagacggagttggaagctccagaattaattcc
Reverse	cttaatgtccacggcatcttgctgttccaagtttaagtcgatttctcg

## Supplementary Materials

The following are available online at http://www.mdpi.com/2221-3759/6/2/9/s1.
