# Supplementary material for: Requirement of the Dynein-Adaptor Spindly for Mitotic and Post-Mitotic Functions in Drosophila
Source: J Dev Biol. 2018 Mar 30;6(2):9. doi: 10.3390/jdb6020009 (PMC6027351; doi:10.3390/jdb6020009)
Supplement: Supplementary file 1 [file jdb-06-00009-s001.zip › Supp_Mat_Clemente_revised/Supplemental Material.pdf]

Supplemental Material

Giuliana D. Clemente, Matthew R. Hannaford, Hamze Beati, Katja Kapp, Jens Januschke, Eric R. Griffis and H.-Arno J. Müller: 'Requirement of the Dynein-adaptor Spindly for mitotic and post-mitotic functions in *Drosophila*'

Suppl. Fig. 1: Western blot analyses of protein lysates of wild-type (wt) and *spindly*[mat67>RNAi (RNAi) embryos. Females were raised and crossed to homozygous *UAS::Spindly*^{RNAi} males at indicated temperatures and 0-3 hours old embryos were collected at 25°C, 21°C and 18°C. RNAi-dependent depletion of anti-Spindly immuno-reactive bands was observed at all temperatures. When using the *mat67::Gal4* driver we did not observe a stringent correlation between the efficacy of RNAi using this driver at different temperatures, probably due to a variable, severe abnormality of early cleavage stages. The intensity of the 100 kDa immuno-reactive band also varied between experiments, probably due to variations in the stability of this presumed degradation product only seen in embryo lysates (anti-tubulin or anti-actin were used as loading control; Mw - Molecular weight (in kDa)).

Suppl. Fig. 2: Expression of mat67>GFP-Spindly in *spindly mat67*>RNAi ovaries. Confocal optical sections through ovarioles expressing full length GFP-Spindly in RNAi knock down background, which were fixed and stained for GFP (green), f-actin (red) and DNA (DAPI, blue). (**A**) distal part of ovariole with germarium (ge) and egg chambers (ec); note that GFP::Spindly is not expressed in the germarium, but strongly expressed in the early egg chambers. **(B)** stage 9 egg chamber; note the accumulation of GFP::Spindly in the posterior pole of the oocyte (ooc). Scale bar: 20 µm.

Movie1
Isolated neuroblast overexpressing UAS>>GFP::Spindly (red) and UAS>>mCherry::tubulin (cyan) under the control of *worniu*>Gal4. Corresponds to figure 1B. GFP::Spindly is recruited to the kinetochore shortly after NEB. At anaphase GFP::Spindly moves away from the metaphase plate towards the spindle pole. GFP::spindly remains at the spindle pole until mitosis is complete whereupon it delocalizes into the cytoplasm. Time stamp: hh:mm. Scale bar: 10 µm.

Movie2
Isolated neuroblast overexpressing UAS>>GFP::Spindly$^{\Delta Ct}$ (red) and UAS>>mCherry::tubulin (cyan) under the control of *worniu*>Gal4. GFP::Spindly$^{\Delta Ct}$ is never recruited to the kinetochore throughout mitosis and remains in the cytoplasm. Corresponds to Figure 1C. Time stamp: hh:mm. Scale bar: 10 µm.

Movie3
Isolated neuroblast overexpressing UAS>>GFP::Spindly$^{\Delta SB}$ (red) and UAS>>mCherry::tubulin (cyan) under the control of *worniu*>Gal4. GFP::Spindly$^{\Delta SB}$ localises similarly to the wild type: it is recruited to the kinetochore following NEB before stripping away from the metaphase plate

towards the spindle pole where it remains until after mitosis. Corresponds to Figure 1D. Time stamp: hh:mm. Scale bar: 10µm.

Movie4
Isolated neuroblast overexpressing UAS>>GFP::Spindly$^{\Delta Nt}$ (red) and UAS>>mCherry::tubulin (cyan) under the control of *worniu*>Gal4. GFP::Spindly$^{\Delta Nt}$ forms cytoplasmic puncta throughout interphase. In mitosis it is recruited to the kinetochore exhibiting dynamics similar to wild type, moving towards the spindle pole at anaphase. GFP::Spindly$^{\Delta Nt}$ returns to cytoplasmic puncta following mitosis. Corresponds to Figure 1E. Time stamp: hh:mm. Scale bar: 10 µm.

Movie5
Isolated neuroblast overexpressing UAS>>GFP::Spindly^{S34A} (red) and UAS>>mCherry::tubulin (cyan) under the control of *worniu*>Gal4. GFP::Spindly$^{\Delta S234A}$ localises similarly to the wild type GFP::Spindly as well as GFP::Spindly$^{\Delta SB}$ localising to the kinetochore after NEB and moving towards the spindle pole upon anaphase onset where it remains until mitosis is complete. Corresponds to Figure 1F. Time stamp: hh:mm. Scale bar: 10µm.

Movie6
Syncytial cleavage division 12 and 13 of an embryo expressing full length GFP::Spindly in *spindly*[mat67>RNAi] background. GFP fluorescence was imaged and single frames were taken at 20 sec intervals and processed with airy scan on a Zeiss LSM 880 confocal microscope. Scale bar: 5 µm.

Movie7
Syncytial cleavage division 12 and 13 of an embryo expressing GFP::Spindly[∆C-term] in *spindly*[mat67>RNAi] background. GFP fluorescence was imaged and single frames were taken at 20 sec intervals and processed with airy scan on a Zeiss LSM 880 confocal microscope. Scale bar: 5 µm.
