# Supplementary figures and images for: Requirement of the Dynein-Adaptor Spindly for Mitotic and Post-Mitotic Functions in Drosophila
Source: J Dev Biol. 2018 Mar 30;6(2):9. doi: 10.3390/jdb6020009 (PMC6027351; doi:10.3390/jdb6020009)

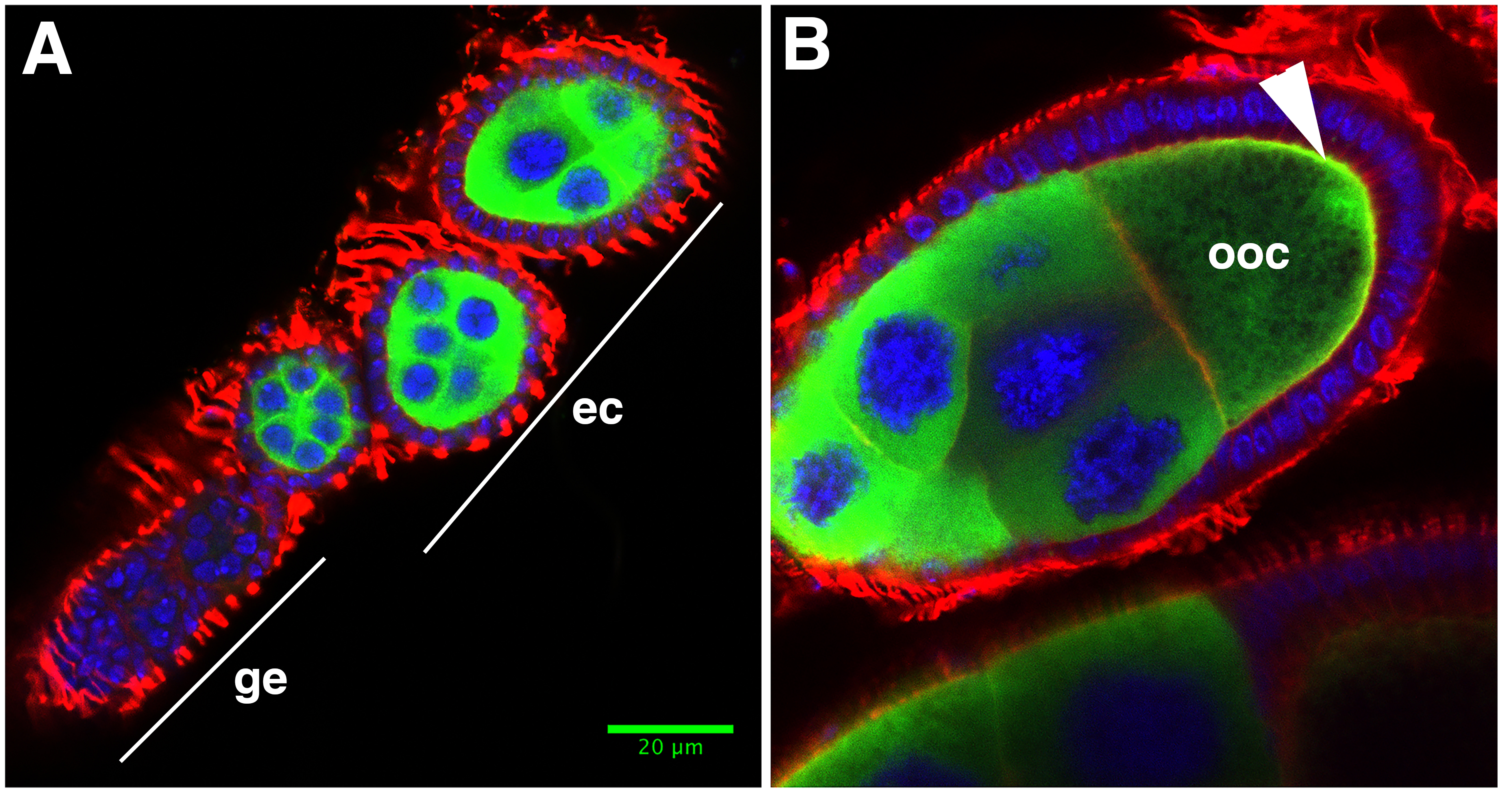

Supplement: Supplementary file 1 [file jdb-06-00009-s001.zip › Supp_Mat_Clemente_revised/Fig_S2.jpg]

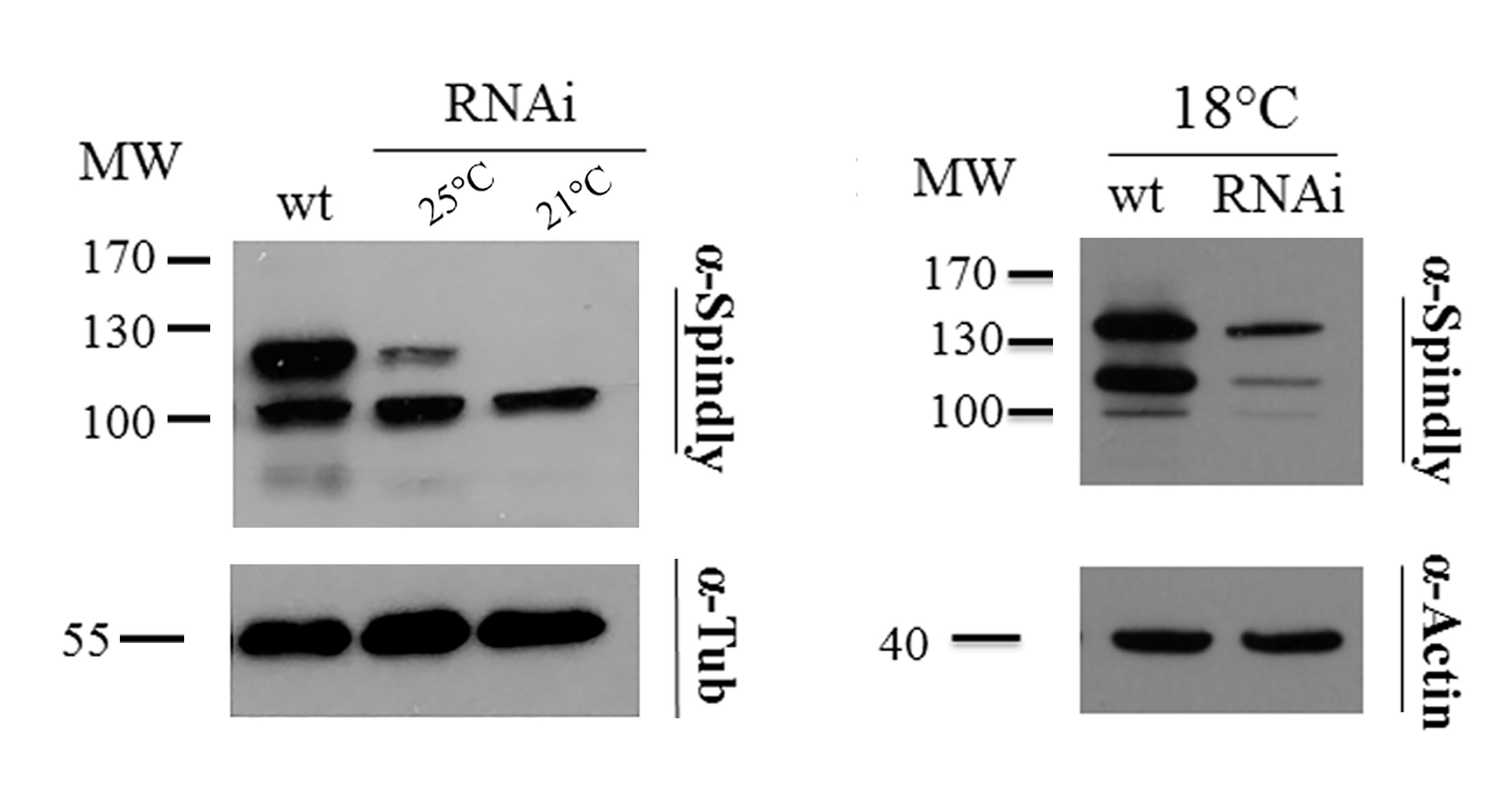

Supplement: Supplementary file 1 [file jdb-06-00009-s001.zip › Supp_Mat_Clemente_revised/Fig_S1.jpg]
